# Detection of Asian genetic components in autochthonous human *Echinococcus multilocularis* infections from endemic Warmia-Masuria (north-eastern Poland)

**DOI:** 10.1016/j.onehlt.2023.100623

**Published:** 2023-08-25

**Authors:** Paweł Gładysz, Anna Lass

**Affiliations:** a Department of Forensic Medicine, Medical University of Gdańsk, Dębowa 23, 80-204 Gdańsk, Poland; b Department of Tropical Medicine and Parasitology, Institute of Maritime and Tropical Medicine, Medical University of Gdańsk, Powstania Styczniowego 9B, 81-519 Gdynia, Poland

**Keywords:** Human alveolar echinococcosis, Poland, Asian origin, Microsatellite EmsB, Mitochondrial sequencing

## Abstract

Alveolar echinococcosis is a life-threatening zoonotic disease caused by the larval stage of the cestode *Echinococcus multilocularis*. People are aberrant intermediate hosts accidentally infected with the parasite eggs via faecal-oral route, usually by the consumption of unwashed fruit and vegetable or direct contact with definitive hosts. The recently reported presence of Asian admixture in *E. multilocularis* tapeworms from Polish red foxes prompted the question of metacestode descent in the human population. In this study, a Maximum Likelihood tree based on partial sequences of *E. multilocularis* mitochondrial genes *cox1*, *cob*, and *nad2* coupled with a hierarchical clustering analysis of microsatellite EmsB profiles and supplemented by Sammon's nonlinear mapping with *k*-means clustering revealed Asian genetic components, to date associated only with the sylvatic cycle, in two autochthonous samples from alveolar echinococcosis patients living in endemic Warmia-Masuria, north-eastern Poland. The red fox is the most likely source of contamination in the environment shared by people and wildlife that led to these infections. Our results confirm that Asian genetic variants participate in the synanthropic cycle in north-eastern Poland and indicate that they may be present in the human population in other areas where Asian genetic variants were detected in red foxes.

## Highlights

• *E. multilocularis* metacestode ancestry in the Euro-Asian contact zone was investigated.
• Microsatellite EmsB and/or mitochondrial genes revealed the Asian origin of two samples.
• Red fox synanthropy introduces Asian variants into the human population.

## 1 Introduction

*Echinococcus multilocularis* Leuckart, 1863 is a parasite widely distributed in the Northern Hemisphere, stretching from western France eastwards to parts of North America. Slow, infiltrative growth of its larval stage in organs characterised by high blood flow is symptomatic of a severe, chronic, and often fatal zoonotic disease, alveolar echinococcosis (AE) [1]. In the sylvatic cycle, the tapeworm circulates between intermediate and definitive hosts; in continental Europe, these roles are played by rodents and wild canids, predominantly the red fox (*Vulpes vulpes* (Linnaeus, 1758)), respectively. Since the human infection occurs via the faecal-oral route, fresh produce contaminated with parasite eggs passed by foxes with stool poses a serious health threat. With oncosphere survival limited by the temperature and humidity of the environment, the geographic range of *E. multilocularis* relies on the mobility and susceptibility of its intermediate and definitive hosts. Dietary interests of migrating foxes affect the involuntary egg deposition in their home range and visited areas.

Between 1980 and 2006, an increase in the red fox population was observed in western and central Poland, resulting from successful rabies vaccination and a change in habitat use following foxes' adaptation to synanthropy [2,3]. Red foxes penetrate agricultural and recreational areas and urban peripheries and risk approaching attractive anthropogenic food sources in rural settings [4]. Individuals visit human settlements to forage fields, farmyards, and allotment gardens and burrow natal dens in abandoned gravel pits, haystacks, railway scarps, and orchards [3].

This pattern has been observed in the hyperendemic Warmia-Masuria Province (north-eastern Poland), as 31.7% of the region is covered by forest, and 54.3% serves as agricultural land (as of 2021) [5]. With a population density of 58 people per km^2^ (41.1% of the Warmian-Masurian population lives in rural areas), scattered households blend into woodlands and fields, promoting human contact with wildlife. Since about half of the red foxes in Warmia-Masuria are infected with the tapeworm (as of 2009–2013, 50.0% (95% CI: 40.3%–59.5%)), there is a high risk of faecal contamination of soil, vegetables, and mushrooms with *E. multilocularis* eggs [[6], [7], [8]]. Indeed, the majority of AE cases in the country come from Warmia-Masuria, though we lack a national register monitoring the epidemiological situation [9].

Notorious genetic homogeneity (a result of extensive homogamy), metacestode polyembryony, a short evolutionary history of the species, and strong selection by a conserved spectrum of hosts make dividing the global population into smaller operational units challenging [10,11]. Numerous attempts have been made to investigate *E. multilocularis* genetic diversity in greater detail. The discriminatory power of mitochondrial and most nuclear markers is limited to the macroregional or continental level but for microsatellite EmsB, which provides resolution on a much finer scale [12,13].

Cladistic analysis of concatenated cytochrome *c* oxidase subunit 1 (*cox1*), cytochrome *b* (*cob*), and NADH dehydrogenase subunit 2 (*nad2*) complete coding sequences using Maximum Likelihood (ML) and Bayesian algorithms revealed a division of *E. multilocularis* into European, Asian, North American, and Mongolian clusters indicating the possibility of isolation during glacial periods, followed by expansion from refugia [13,14]. Using a rapid substitution rate of 10% divergence per million years, the bifurcation of European and Asian clades was estimated to have occurred 60,000–37,000 years ago. After the Last Glacial Maximum, isolated populations recolonised Europe via red foxes migrating from a historical endemic area in Switzerland and the Swabian Jura through Czechia and Slovakia to southern Poland and further up north, to Latvia and Estonia, spreading the parasite fast enough to mitigate genetic drift [15]. Simultaneously, Asian survivors spread westward from Beringia [12].

The two currents meet in Eastern Europe, though data from Kaliningrad Oblast, Lithuania, Belarus, and Ukraine is insufficient to characterise the nature of this sympatry in detail; the influence from Lithuanian foxes through Russia onto Warmia-Masuria and further into eastern Poland southward has been hypothesised [15,16]. A nationwide study of *E. multilocularis* genetic diversity in Poland based on concatenated *cox1*, *cob*, and *nad2* complete coding sequences revealed the presence of three out of fifteen Polish mitochondrial haplotypes in Warmia-Masuria. One of them, found only in north-eastern Poland, clustered with haplotypes from Japan and Kazakhstan [17]. Further study based on *cox1* sequencing and EmsB profiling confirmed the presence of tapeworms of Asian descent in five Polish provinces (Warmia-Masuria, Podlasie, Mazovia, Kujawy-Pomerania, and Lubuskie bordering the state of Brandenburg, Germany) and provided evidence for cross-fertilisation between individuals of Asian and European lines [18]. However, such metacestodes infecting European people have yet to be reported.

Co-habitation of red foxes and people is conducive to the penetration of foreign genotypes already present in wild definitive hosts into the human population. Their impact on human health is yet unknown. Using phylogenetic inference and microsatellite profiling, we test the hypothesis that Asian variants of *E. multilocularis* have entered the synanthropic life cycle in Warmia-Masuria, a hyperendemic region of north-eastern Poland.

## 2 Material and methods

### 2.1 Samples

DNA was extracted from thirteen samples collected between 1998 and 2020 from patients of the University Centre for Maritime and Tropical Medicine in Gdynia treated for AE who have never travelled to Asia and live in endemic Warmia-Masuria Province (except for sample 1403, which was a DNA isolate from the collection of Beata Szostakowska, PhD, Division of Tropical Parasitology, Medical University of Gdańsk) (Table 1). The region is well-recognised in terms of the epidemiology of AE [9], and *E. multilocularis* prevalence in red foxes has been documented there since 1995 [6]. As of 2008, 45.5% of canids were infected with the tapeworm in the district of Lidzbark (11 foxes examined), 55.9% in Kętrzyn (34 foxes examined), 22.2% in Węgorzewo (9 foxes examined), and 37.5% in Olsztyn (56 foxes examined) [31]. No positive foxes were detected in the districts of Elbląg and Giżycko; however, in these areas, only four and two foxes were tested, respectively. The sampled patients aged 30–71 (median age 51) exhibited mild to advanced disease stages II–IV [19].

**Table 1:** Samples from patients of the University Centre for Maritime and Tropical Medicine in Gdynia treated for alveolar echinococcosis (AE) and living in Warmia-Masuria (north-eastern Poland) with PNM assignment and respective disease stages. The two indicators form a system of clinical classification for AE introduced by the European Network for Concerted Surveillance of AE and the WHO Informal Working Group on Echinococcosis. Partial sequences of all three mitochondrial genes were obtained for samples marked with an asterisk. P – parasitic mass in the liver, N – involvement of neighbouring organs, M – metastasis. Sample 1403 was a DNA isolate from the collection of Beata Szostakowska, PhD, Division of Tropical Parasitology, Medical University of Gdańsk.

Sample	District	Sex	Age	PNM	Stage	Collection date	Type	Preservative	Storage conditions
525*	Węgorzewo	M	30	P3N0M0	IIIa	24/07/2001	metacestode in liver	none	-20 °C
795*	the city of Elbląg	M	36	P2N0M0	II	08/06/2005	metacestode in liver	ethanol solution 70%	room temp.
853*	Lidzbark	F	30	P2N0M1	IV	07/08/2006	metacestode in liver	ethanol solution 75%	room temp.
1403*	Giżycko	M	57	P3N0M1	IV	unknown	unknown	N/A	-20 °C
1664*	Kętrzyn	F	63	P2N0M0	II	20/08/2014	fluid drained from liver	ethanol solution	room temp.
1857*	the city of Olsztyn	M	32	P2N0M1	IV	17/03/2017	metacestode in pancreas	ethanol solution	room temp.
80	Lidzbark	M	31	P3N0M1	IV	03/09/1998	metacestode in liver	none	-20 °C
843	Elbląg	M	58	P2N0M0	II	03/04/2006	unknown	ethanol solution 95%	room temp.
855	Lidzbark	M	48	P3N0M0	IIIa	07/08/2006	metacestode in liver	formaldehyde solution 4%	room temp.
863	Bartoszyce	K	68	P2N0M0	II	26/10/2006	metacestode in liver	ethanol solution 70%	room temp.
1153	Szczytno	M	51	P4N1M0	IIIb	03/04/2010	fluid drained from liver	none	-20 °C
1635	Olsztyn	K	71	P2N0M0	II	09/04/2014	metacestode in liver	ethanol solution	room temp.
1925	Szczytno	K	52	P2N1M0	IIIb	14/08/2018	metacestode in liver	ethanol solution	room temp.

Extraction from the liver/pancreas metacestode or drained fluid was performed using Genomic Mini AX Tissue (A&A Biotechnology, Gdańsk, Poland, cat. no. 056–60), an increased efficiency kit for genomic DNA purification, according to the manufacturer's protocol. Due to the poor quality of templates, partial sequences of all three mitochondrial genes were obtained from only six samples (Table 1).

The exact concentration of metacestode DNA in isolates admixed with patients' DNA was unknown, rendering quantification for PCR impossible. Additionally, templates were expected to be of poor quality due to long-term storage conditions of the source material (room temperature, tissues only partially submerged in ethanol) and, in the case of sample 1403, due to degradation caused by frequent freezing and thawing over the course of five years.

### 2.2 Mitochondrial sequencing

Partial sequences of *cox1*, *cob*, and *nad2* mitochondrial genes were amplified using a varied amount of total isolated DNA (114–1865 ng) per total reaction volume of 25 μl, 12.5 μl of DreamTaq Green PCR Master Mix (2×) (Thermo Scientific™), and primers added to a final concentration of 0.4 μM (Table 2). Amplicons were purified enzymatically with EPPiC Fast (A&A Biotechnology, Gdańsk, Poland, cat. no. 1021-100F) and outsourced to Macrogen Europe (Amsterdam) for bidirectional sequencing.

**Table 2:** PCR primers and conditions for the amplification of microsatellite EmsB and partial sequences of cytochrome *c* oxidase subunit 1 (*cox1*), cytochrome *b* (*cob*), and NADH dehydrogenase subunit 2 (*nad2*) genes.

Marker	Primers (5′ → 3′)	Expected amplicon size [bp]	Source	PCR conditions
*cox1*	F: TTTACTTTGGATCATAAGCG R: CCAAAAAACCAAAACATATGTTGAA	638–668	[29]	95 °C — 4 min; 35 cycles: 95 °C — 1 min, 48 °C — 1 min, 72 °C — 1 min; 72 °C — 10 min
*cob*	F: GTTTAAACTGGTAGATTGTGGTTC R: CTCCACAGTAGAAATCACCATCA	1323	[30]	95 °C — 4 min; 35 cycles: 95 °C — 30 s, 50–52 °C — 30 s, 72 °C — 60–90 s; 72 °C — 10 min
*nad2*	F: GCGTTGATTCATTGATACATTGT R: TAGTAAAGCTCAAACCGAGTTCT	1032	[13]	95 °C — 4 min; 35 cycles: 95 °C — 30 s, 52 °C — 30 s, 72 °C — 90 s; 72 °C — 10 min
EmsB	F: (FAM)—GTGTGGATGAGTGTGCCATC R: CCACCTTCCCTACTGCAATC	209–241	[10]	95 °C — 3 min; 35 cycles of 95 °C — 30 s, 60 °C — 1 min, 72 °C — 1 min; 72 °C — 30 min

Regions on 5′ and 3′ ends with more than a 1% chance of an error per base were automatically trimmed, and forward and reverse reads were assembled in Geneious (https://www.geneious.com/). Consensus sequences were checked for internal stop codons to ensure orthologs were amplified. The species was confirmed with BLAST [20].

For each marker, the six samples were supplemented with fifteen sequences representing Polish haplotypes, including EmPL9 of confirmed Asian origin (KY205662–KY205706) [17,18], and eight sequences representing European, Asian, and North American genotypes retrieved from GenBank (AB461395–AB461420AB461395AB461396AB461397AB461398AB461399AB461400AB461401AB461402AB461403AB461404AB461405AB461406AB461407AB461408AB461409AB461410AB461411AB461412AB461413AB461414AB461415AB461416AB461417AB461418AB461419AB461420) [13]. The datasets were aligned using the MUSCLE algorithm in MEGA11 [21] and concatenated to a total of 2377 bp (*cox1*: 555 bp, *cob*: 1003 bp, *nad2*: 819 bp).

**Fig. 1:**
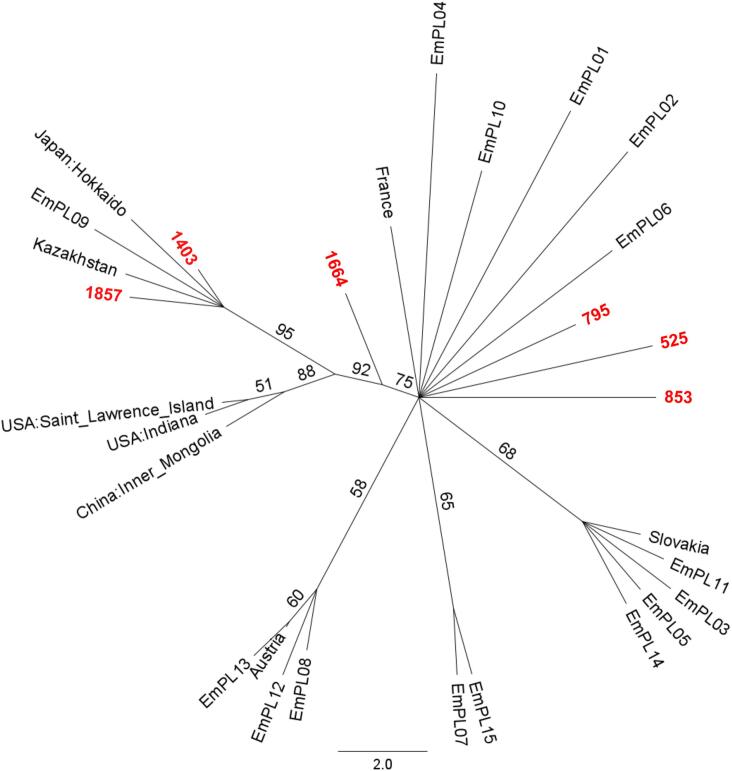
Maximum Likelihood unrooted consensus tree, built with the support threshold set to 50% and 25% burn-in, based on concatenated partial coding sequences of *cox1* (555 bp), *cob* (1003 bp), and *nad2* (819 bp) mitochondrial genes (a total of 2377 bp). Apart from the six studied samples (in red), the dataset comprised fifteen sequences representing Polish haplotypes, including EmPL9 of confirmed Asian origin (KY205662–KY205706) [17,18], and eight sequences representing European, Asian, and North American haplotypes (AB461395–AB461420) [13]. Partitioned analysis was performed by implementing the GTR CAT nucleotide model. Values above branches represent bootstrap support in % (1000 replicates).

Partitioned analysis was performed using the RAxML version 8.2.11 [22] plugin in Geneious by implementing the GTR CAT nucleotide model and calculating branch support by bootstrapping (rapid hill-climbing; random seed = 1; 1000 replicates). A consensus tree was built with the support threshold set to 50% and 25% burn-in (Fig. 1).

### 2.3 Hierarchical clustering analysis

Prior to fragment size analysis, synthesis and cloning of a calibrating sequence specified in [23] were ordered. Capillary electrophoresis of the calibrator revealed a 2-bp shift between the expected and observed allele length. Thus, when processing the results, each peak size was increased by 2 bp and reduced by 1 bp to correct for the adenylation of products by DNA polymerase.

For each sample, two independent PCR reactions were performed in a 25-μl reaction mixture containing a varied amount of total isolated DNA (205–2352 ng), 12.5 μl of PCR Mix Plus HGC (A&A Biotechnology, Gdańsk, Poland, cat. no. 2005–100G), and primers added to a final concentration of 0.4 μM (Table 2). Capillary electrophoresis of PCR products was performed on an Applied Biosystems® 3130 Genetic Analyzer (Life Technologies, Foster City, CA), and the results were analysed in GeneMapper 3.1. Profiles were processed and normalised according to [23].

To assert reproducibility and ensure that all alleles were amplified, two indicators were calculated for each pair of resulting profiles, namely Euclidean distance and Spearman's rank correlation coefficient (Table 3). Profiles were considered significantly similar if d < 0.08, i.e., below the genetic distance threshold employed in the hierarchical clustering analysis [10]. Five samples met this requirement. Due to low peak heights and possible stochastic effects at play, the PCR of sample 1403 was repeated with an increased volume of template, resulting in a vastly different profile. This could mean that due to a low quantity and/or quality of metacestode nuclear DNA, the result did not reflect the true structure of the EmsB region in chromosome 5. Because the volume of the isolate was insufficient to proceed with reliable profiling, sample 1403 was discarded from fragment size analysis.

**Table 3:** Samples from patients of the University Centre for Maritime and Tropical Medicine in Gdynia treated for alveolar echinococcosis (AE) and living in Warmia-Masuria (north-eastern Poland) with the fluorescence intensity of the obtained EmsB profiles and values of Euclidean distance (d) and Spearman's rank correlation coefficient (ρ; *p* < 0.001 except for *) as estimates of profiling reproducibility. From each pair of profiles, the one characterised by a higher normalisation 10% cut-off value of fluorescence intensity was selected for hierarchical clustering analysis; sample 1403 was discarded. Sample 1403 was a DNA isolate from the collection of Beata Szostakowska, PhD, Division of Tropical Parasitology, Medical University of Gdańsk.

Sample	Profile 1		Profile 2		d	ρ
	Fluorescence intensity	Normalisation 10% cut-off value	Fluorescence intensity	Normalisation 10% cut-off value		
525	18,986	305.0	13,491	281.1	0.0634	0.96
795	29,477	467.2	10,706	157.5	0.0255	0.98
853	22,225	287.6	8,669	117.7	0.0630	0.92
1664	24,366	474.9	31,250	470.3	0.0680	0.96
1403	3,595	156.2	9,085	377.0	0.7782	−0.30*
1857	23,097	455.2	9,561	221.2	0.0609	0.97

From each of the remaining five pairs of profiles, the profile characterised by a higher normalisation 10% cut-off value of fluorescence intensity was pooled with data from EmsB Website for Echinococcus Typing (https://ewet-db.univ-fcomte.fr/) representing Europe, Asia (Kyrgyzstan: Alay Valley, Japan: Hokkaido, China: Shiqu County), and the Arctic (USA: St. Lawrence Island, Canada: Nunavut n. Manitoba, Norway: Svalbard). The set was supplemented with Polish profiles of Asian origin [16,18].

Because marker EmsB offers high resolution suitable for microregional studies, Europe was represented only by profiles from Warmia-Masuria, both EWET records and data not yet uploaded [16,18]. If the tested samples are native to Warmia-Masuria, they will cluster together. Should they represent other European variants, they will form (a) separate grouping(s) with Polish samples. Only a strong resemblance to one of the Asian profiles, indicative of considerable genetic proximity, would allow the UPGMA algorithm to place a sample in the Asian cluster.

**Fig. 2:**
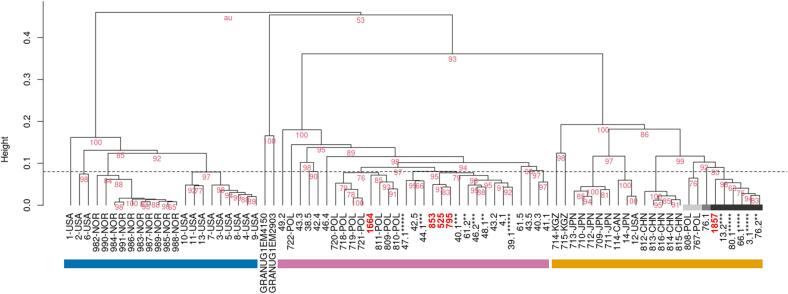
Phenogram constructed using obtained EmsB profiles (in red), data from the EWET repository, and profiles not yet uploaded (Umhang et al., 2017) with *Echinococcus granulosus* (G1) profiles GRANUG1EM4150 and GRANUG1EM2903 introduced as species controls. Tapeworms with the same profile extracted from one fox were pooled (the no. of asterisks corresponds to the no. of parasites). The approximately unbiased *p*-values (in % in red) were calculated with a multiscale bootstrap resampling (1000 replicates). Leaf-node colours: light grey – APol1, medium grey – APol2, dark grey – APol3 (cf. Umhang et al., 2021b). Colour bars: blue – North America and the Arctic, pink – Warmia-Masuria, orange – Asia. (For interpretation of the references to colour in this figure legend, the reader is referred to the web version of this article.)

**Fig. 3:**
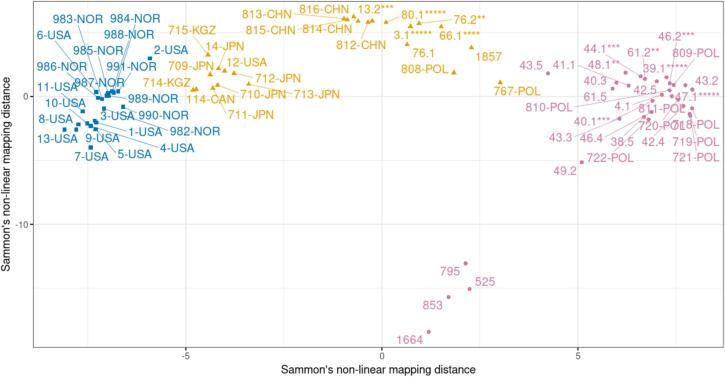
Grouping of obtained EmsB profiles, data from the EWET repository, and profiles not yet uploaded (Umhang et al., 2017) by Sammon's nonlinear mapping with *k*-means. Tapeworms with the same profile extracted from one fox were pooled (the no. of asterisks corresponds to the no. of parasites). Colours: blue – North America and the Arctic, pink – Warmia-Masuria, orange – Asia. (For interpretation of the references to colour in this figure legend, the reader is referred to the web version of this article.)

Hierarchical clustering analysis using Euclidean distance and unweighted pair group method with arithmetic mean [24] was performed using R version 4.2.3 [25] (Fig. 2). The stability of clusters was assessed by a multiscale bootstrap resampling (1000 replicates) and given as approximately unbiased *p*-values [26,27]. To avoid over-discrimination of variability, a genetic distance threshold of 0.08 was used to delimit the interpretation of bifurcations [10]. The results were validated with Sammon's nonlinear mapping with *k*-means clustering under the assumption that there were three clusters (Fig. 3).

## 3 Results

### 3.1 Mitochondrial sequencing

In the ML tree (Fig. 1), samples 795, 525, and 853 grouped with European sequences (moderate branch support of 75%). The genetic origin of sample 1664 is inconclusive. Samples 1403 and 1857 clustered with sequences from Japan and Kazakhstan within the Asian-American clade (92%) with strong bootstrap support of 95%.

### 3.2 Hierarchical clustering analysis

Sample 1664 from the district of Kętrzyn clustered with EWET profiles of tapeworms extracted from red foxes from the neighbouring district of Mrągowo (Fig. 2). Samples 525, 853, and 795 formed a strong grouping with isolates from seven different districts of Warmia-Masuria (the European-nuclear/Asian-mitochondrial hybrid 40.1 among them). The algorithm unambiguously ascribed all four samples to the European clade, as indicated by the pink bar in Fig. 2.

Olsztyn sample 1857 clusters with isolates from five Polish provinces: Lubuskie (13.2), Mazovia (80.1), Podlasie (66.1), Kujawy-Pomerania (76.2), and the Warmian-Masurian sample 3.1 from the district of Ostróda, an area adjacent to the district of Olsztyn surrounding the city of Olsztyn. Together, they form a clade in the Asian section of the phenogram, as indicated by the orange bar in Fig. 2.

### 3.3 Sammon's nonlinear mapping with *k*-means clustering

The mapping was performed to validate the results of the hierarchical clustering analysis. With the assumption of three clusters, the resulting profile groups corresponded to the main branches of the UPGMA phenogram. Samples 525, 795, 853, and 1664 belong to the European part of the diagram (pink grouping in Fig. 3), whereas sample 1857 distinctly clusters with Polish Asian and Chinese samples in the Asian cloud of points (orange grouping in Fig. 3).

## 4 Discussion

Investigation of three mitochondrial genes and microsatellite profiling revealed Asian genetic input in two samples of metacestode DNA, namely 1403 and 1857, obtained from Warmian-Masurian AE patients who have never been to Asia. However, without the profile for sample 1403, mitochondrial genotyping alone provides limited evidence of its origin. It is possible that this metacestode's nuclear genome was partly European as a result of male introgression, depending on the hybrid generation [18].

As for sample 1857, there is evidence for the Asian origin of mitochondrial and nuclear genomes. The previously defined grouping of Polish Asian profiles comprises three red-fox *E. multilocularis* metaprofiles APol1–3 demarcated by the standard genetic distance threshold of 0.08 in the study conducted by Umhang et al. (2021) [18]. It is worth noting that samples 13.2 and 80.1 are confirmed Asian-nuclear/European-mitochondrial hybrids, and samples 3.1, 66.1, and 76.2 are of pure Asian descent [18]. The red fox is the most likely source of contamination that led to the infection of patient 1857.

The entire grouping APol1–3 (indicated in Fig. 2 by grey bars) is genetically too distant from other Polish isolates to cluster within the European clade but, more importantly, too similar to Chinese samples from intermediate hosts *Microtus limnophilus* Büchner, 1889 and *Cricetulus kamensis* (Satunin, 1903) [10] to allow for a continental-level misplacement in the phenogram. APol1–3 near-Chinese provenance is further supported by Sammon's nonlinear mapping with *k*-means clustering (in orange in Fig. 3), where it forms a distinct cloud of points with the samples from Shiqu County (Sichuan Province), an area located about 7000 km away from Poland.

When it comes to the epidemiological relevance of these findings, an important distinction has to be made about tools used to assess the genetic diversity of parasitic tapeworms. So far, no correlation has been found between particular EmsB profiles and tapeworms' pathogenicity/virulence. The current state of the art is that, as a geographically-relevant marker, EmsB does not inform us on pathogenicity/virulence. Based on the degree of profile similarity, it fine-grains the observed *E. multilocularis* distribution and allows us to hypothesise about populations' post-glacial movement and recent evolutionary history. Testing for correlation between EmsB polymorphism and AE severity would require a considerably more extensive dataset, including patients' medical history. Relevant studies based on human samples are limited to 63 cases dating 1981–2019 from France, Switzerland, Germany, and Belgium, but the analysis of association between particular profiles, affected organs, and number of lesions was aborted due to a limited availability of the PNM data [28]. No such research has been conducted in the investigated area. In fact, AE in Warmia-Masuria may be significantly underdiagnosed. So far, few tapeworms extracted from Warmian-Masurian foxes have been profiled, revealing a fraction of the estimated diversity [16].

A biologically-relevant marker strictly correlated with the cestode's (in)ability to thrive in the human organism would be best for AE control and prevention. In the age of genome-wide association studies, several markers collectively responsible for the cestode's pathogenicity/virulence will likely be identified.

## 5 Conclusions

Phylogenetic investigation of three mitochondrial markers and hierarchical clustering analysis of EmsB profiles revealed Asian genetic components in two autochthonous metacestode samples from AE patients living in endemic Warmia-Masuria (north-eastern Poland), who have never travelled to an Asian country. Fox-related egg contamination in the environment shared by people and wildlife is a key factor in the penetration of foreign variants, to date associated only with the sylvatic cycle, into the human population.

## Funding

This work was supported by the 10.13039/501100004281National Science Centre grant no. 2020/37/B/NZ7/03934.

## CRediT authorship contribution statement

**Paweł Gładysz:** Conceptualization, Methodology, Software, Validation, Formal analysis, Investigation, Data curation, Writing – original draft, Writing – review & editing, Visualization. **Anna Lass:** Resources, Writing – review & editing, Supervision, Project administration, Funding acquisition.

## Declaration of Competing Interest

The authors declare no competing interests.

## Data Availability

Mitochondrial sequences obtained in this study are available in GenBank under accessions OQ595100–OQ595105 (*cox1*); OQ616346, OQ616350, OQ616351, OQ616354, OQ616356, OQ616357 (*cob*); OQ616331, OQ616335, OQ616337, OQ616340, OQ616342, OQ616343 (*nad2*). Raw FSA files and normalised EmsB profiles of metacestodes extracted from patients of the University Centre for Maritime and Tropical Medicine in Gdynia treated for alveolar echinococcosis and living in Warmia-Masuria (north-eastern Poland), R script to calculate and visualise a UPGMA phenogram with a genetic distance threshold, and R script to perform Sammon’s nonlinear mapping with k-means clustering are available at Mendeley Data, doi: 10.17632/45gp9scht8.1.
